# Involvement of hormones in olfactory imprinting and homing in chum salmon

**DOI:** 10.1038/srep21102

**Published:** 2016-02-16

**Authors:** Hiroshi Ueda, Shingo Nakamura, Taro Nakamura, Kaoru Inada, Takashi Okubo, Naohiro Furukawa, Reiichi Murakami, Shigeo Tsuchida, Yonathan Zohar, Kotaro Konno, Masahiko Watanabe

**Affiliations:** 1 Field Science Center for Northern Biosphere, Hokkaido University, Sapporo 060-0809, Japan; 2 Division of Biosphere Science, Graduate School of Environmental Science, Hokkaido University, Sapporo 060-0809, Japan; 3 Department of Natural History Sciences, Graduate School of Science, Hokkaido University, Sapporo 060-0810, Japan; 4 Department of Marine Biotechnology, Institute of Marine and Environmental Technology, University of Maryland, Baltimore, MD 21202, USA; 5 Department of Anatomy, Graduate School of Medicine, Hokkaido University, Sapporo 060-8638, Japan

## Abstract

The olfactory hypothesis for salmon imprinting and homing to their natal stream is well known, but the endocrine hormonal control mechanisms of olfactory memory formation in juveniles and retrieval in adults remain unclear. In brains of hatchery-reared underyearling juvenile chum salmon (*Oncorhynchus keta*), thyrotropin-releasing hormone gene expression increased immediately after release from a hatchery into the natal stream, and the expression of the essential NR1 subunit of the N-methyl-D-aspartate receptor increased during downstream migration. Gene expression of salmon gonadotropin-releasing hormone (sGnRH) and NR1 increased in the adult chum salmon brain during homing from the Bering Sea to the natal hatchery. Thyroid hormone treatment in juveniles enhanced NR1 gene activation, and GnRHa treatment in adults improved stream odour discrimination. Olfactory memory formation during juvenile downstream migration and retrieval during adult homing migration of chum salmon might be controlled by endocrine hormones and could be clarified using NR1 as a molecular marker.

Salmon migration is iconic in ecology and is also culturally and economically important to many individuals; however, migratory salmon are under threat globally from overharvesting, anthropogenic pollution, climate change, and environmental degradation^1^,^2^,^3^,^4^. To date, there are no useful management methods for salmon conservation and production based on the success of salmon migration.

The olfactory hypothesis for salmon homing to their natal stream was proposed in the 1950s^5^,^6^, and the mechanisms of olfactory imprinting and homing in salmon have been intensively researched using behavioural, electrophysiological, biochemical, and neurobiological methods^7^,^8^,^9^. Many studies have also explored the hormonal control mechanisms of juvenile downstream migration and adult homing migration in relation to seawater adaptability^10^,^11^ and gonadal maturation^12^, respectively. However, until now, it has been impossible to link hormonal control mechanisms to imprinting and homing because we lacked molecular markers that would permit the evaluation of olfactory memory formation and retrieval in the salmon brain.

The N-methyl-D-aspartate receptor (NMDAR) is a glutamate receptor channel subtype and mediates most of the fast excitatory synaptic transmission in the central nervous system. It plays important roles in memory formation and retrieval in higher vertebrates^13^,^14^ and in fish^15^,^16^,^17^,^18^,^19^. NMDARs are comprised of two subunits: the essential NR1 subunit and the differentially expressed NR2A-D subunit^20^. The NR1 gene of chum salmon (*Oncorhynchus keta*) has recently been cloned and characterized^21^, and the effects of changing salinity on NR1 gene expression have been reported^22^. However, there have been no reports addressing how NR1 might be involved in olfactory memory formation and retrieval in salmon. The thyroid hormones have been reported to play important roles in juvenile chum salmon during downstream migration^23^ and in olfactory cellular proliferation in coho salmon (*O. kisutch*) during a sensitive period for imprinting^24^. Gonadotropin-releasing hormone (GnRH) is considered a leading hormone for salmon homing migration^12^, and implantation of GnRH analogue (GnRHa) was able to shorten the homing duration of lacustrine sockeye salmon (*O. nerka*) in Lake Shikotsu (Hokkaido, Japan)^25^,^26^.

Here, we show that the gene expression of NR1 and thyrotropin-releasing hormone (TRH) increased during downstream migration to the sea in the juvenile chum salmon brain and that NR1 and salmon gonadotropin-releasing hormone (sGnRH) gene expression increased in the brain during adult homing from the Bering Sea to the natal hatchery. Juvenile treatment with thyroid hormone enhanced NR1 gene activation, and adult treatment with GnRHa increased stream odour discrimination ability. This work demonstrates that NR1 expression in the brain plays crucial roles in olfactory memory formation and retrieval that might be controlled by endocrine hormones in juvenile and adult chum salmon, respectively. Investigation of this mechanism will allow researchers to predict responses to altered biochemical environments and provides tools to explain migratory salmon behaviour, including straying rates.

## Results

**Figure 1:**
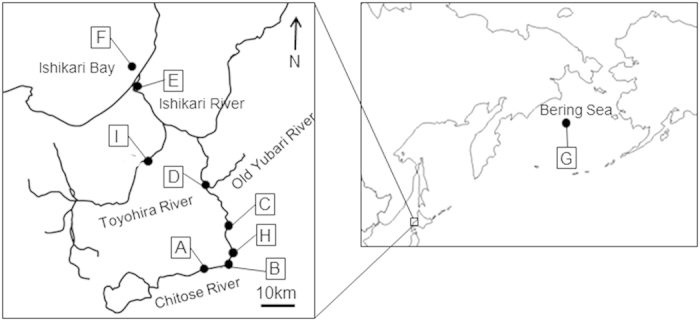
Map showing the study area with the sampling sites of juvenile chum salmon during downstream migration, adult chum salmon during homing migration, and river water for electro-olfactogram experiments. (A): Chitose Hatchery; (B): second bridge of the Chitose; (C): Chitose River in Kamaka Ward, Chitose; (D): Confluence point of the Chitose and Yubari Rivers; (E): Mouth of the main stem of the Ishikari River; (F): Ishikari Bay; (G): Bering Sea; (H): Indian Waterwheel, Chitose River. River water sampling points were the second bridge of the Chitose River (B), the Toyohira River (I), and the mouth of the Ishikari River (E). This map was created using Adobe Illustrator CS4.

Using underyearling juvenile chum salmon reared in a hatchery and then released into the Chitose River (Hokkaido, Japan) to allow migration to the sea (Fig. 1), the relationship between brain-pituitary-thyroid (BPT) hormones and natal stream odour imprinting was investigated based on whole-brain mRNA expression of thyrotropin-releasing hormones (TRHa and TRHb; neurohormone in the salmon brain and the leading hormone in the BPT axis) and NR1, upper head (containing the pituitary gland) thyrotropin β subunit (TSHβ), thyroid hormones (thyroxine (T4) and triiodothyronine (T3)) isolated in the lower jaw that contained thyroid glands, and the electro-olfactogram response (EOG) to stream water odour.

**Figure 2:**
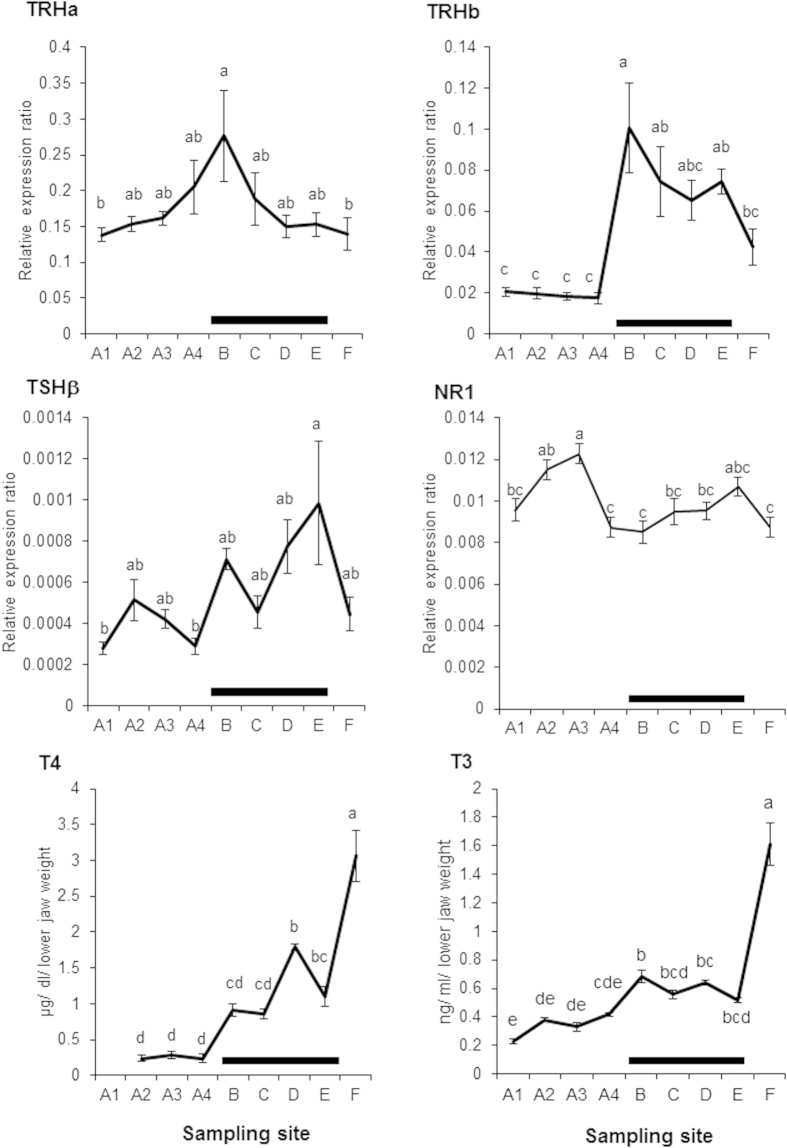
Changes in the profiles of the brain-pituitary-thyroid hormones and the N-methyl-D-aspartate receptor essential subunit NR1 in juvenile chum salmon during downstream migration. Fish were sampled at the Chitose Hatchery (Site **A**) at four time points (January (A1), February (A2), March (A3) and April (A4)), and following release into the Chitose River at the second bridge of the Chitose (Site **B**), the Chitose River in Kamaka Ward (Site **C**), the confluence of the Chitose and Yubari Rivers (Site **D**), the mouth of the Ishikari River (Site **E**), and Ishikari Bay (**F**). The gene expression ratios of thyrotropin-releasing hormone, TRHa and TRHb in the whole brain, TSHβ in the upper-head, and NR1 in the whole brain, were normalized to the reference gene (β-actin), and the expression levels were compared using the relative Ct (ΔΔCT) method. The contents of thyroxine (T4) and triiodothyronine (T3) in the lower jaw are expressed in μg/dl/lower jaw weight and ng/dl/lower jaw weight, respectively. Data are presented as the means ± SEM (N = 10–15). Different letters represent significant differences using the Tukey-Kramer multiple comparison test (TRHa, TRHb, TSHβ, NR1, and T3) and Scheffe’s F test (T4) (*p* < 0.05). The horizontal bar indicates the locations passed during juvenile downstream migration to the bay.

TRHa gene expression increased gradually during rearing in the hatchery and peaked significantly immediately after release into the Chitose River at Site **B** (Fig. 2). In contrast, TRHb gene expression showed no changes in the hatchery but a significant increase after release at Site **B** (Fig. 2). Although the precise roles of TRHa and TRHb are unknown, TRHb appears to react to acute environmental changes, such as release into the river. TSHβ gene expression showed a gradual increase at Site **B** and peaked significantly at Site **E**. T4 and T3 levels then increased significantly at Site **F** (Fig. 2). These data support the importance of the BPT axis in the early downstream migratory period of chum salmon and are consistent with the hypothesized role of thyroid hormones during the imprinting phase in coho salmon^24^.

**Figure 3:**
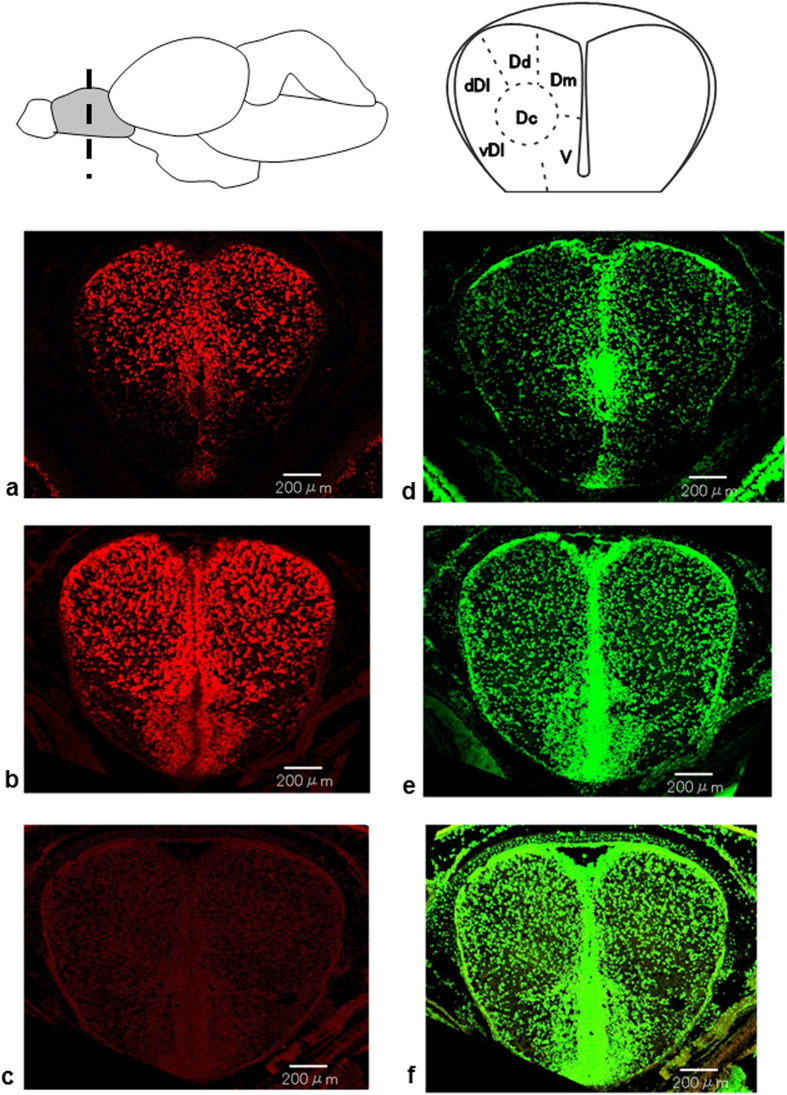
Laser-scanning confocal microscope images showing the distribution of N-methyl-D-aspartate receptor essential subunit NR1 mRNA in the telencephalon of juvenile chum salmon. Fish were sampled at the Chitose Hatchery (Site **A**; **a**) and the Chitose River in Kamaka Ward (Site **C**; **b,c**) in April 2013. (**c)** Sence cRNA probe as a control. Sections were counterstained with Nissl stain (**d**: Site **A**; **e,f**: Site **C**). The dashed line indicates the coronal plane cut. Abbreviations: dDl, dorsal part of lateral area of dorsal telencephalon (TE); vDl, ventral part of lateral area of dorsal TE; Dd, dorsal part of dorsal TE; Dc, central area of dorsal TE; Dm, medial area of dorsal TE; V, ventral area. Scale bar, 200 μm.

The gene expression of NR1 in juveniles peaked in March, when larval brain development became prominent^27^, and then declined during rearing in April in the hatchery (Fig. 2). NR1 expression began to increase significantly at Site **C**, following the significant increase in TRHa/b gene expression at Site **B**, and showed a second peak at Site **E** (Fig. 2). *In situ* hybridization localized NR1 mRNA to the dorsolateral telencephalon (TE) when the fish were in the hatchery. Expression was also prominent in the ventral TE, which is connected with the olfactory bulb (OB)^28^, following hatchery release in fish captured at Site **C** (Fig. 3).

**Figure 4:**
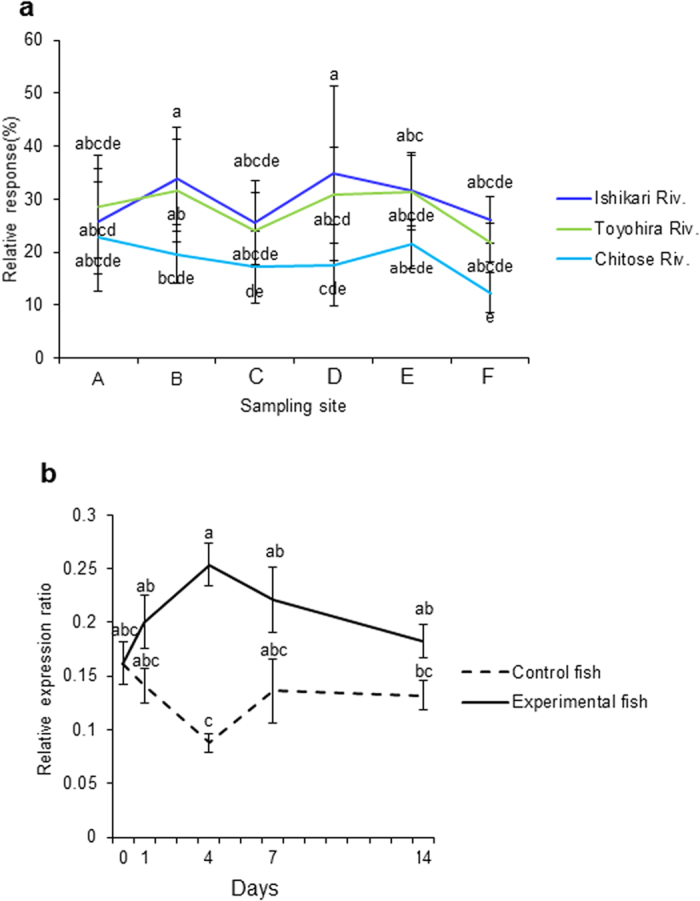
(**a**) Electro-olfactogram (EOG) responses to waters from three streams in juvenile chum salmon during downstream migration. (**b**) Effects of oral thyroxine (T4) administration on the gene expression ratios of the N-methyl-D-aspartate receptor essential subunit NR1 in the whole brain of juvenile chum salmon. (**a**) Juvenile fish were sampled at the Chitose Hatchery (Site **A**), the second bridge of the Chitose (Site **B**), the Chitose River in Kamaka Ward (Site **C**), the confluence point of the Chitose and Yubari Rivers (Site **D**), the mouth of the Ishikari River (Site **E**), and Ishikari Bay (Site **F**). The values are the means ± SEM of data obtained from three juvenile fish. Different letters represent significant differences using one-way ANOVA followed by the Dunnett test (*p* < 0.05). (**b**) Juvenile chum salmon reared in the Chitose Hatchery were transferred to the Toyohira Salmon Museum, fed pellets containing 2 mg/g T4 (Experimental fish) or pellets without T4 (Control fish), and sampled at days 0, 1, 4, 7, and 14 to evaluate NR1 expression. The gene expression levels of NR1 in the whole brain were normalized to the reference gene (β-actin), and the expression levels were compared using the relative Ct (ΔΔCT) method. Data are presented as the means ± SEM (N = 7–8). Different letters present significant differences using the Steel-Dwass test (*p* < 0.05).

The olfactory discrimination ability of stream odours, measured by EOG, showed significantly greater discrimination ability for Ishikari River (home stream) water in juvenile chum salmon collected at Sites **B** and **D** compared to the fish at the hatchery (Fig. 4a). These data suggest that the capacity for forming olfactory memory of stream odours continues and may increase during the downstream migration of juvenile chum salmon.

**Figure 5:**
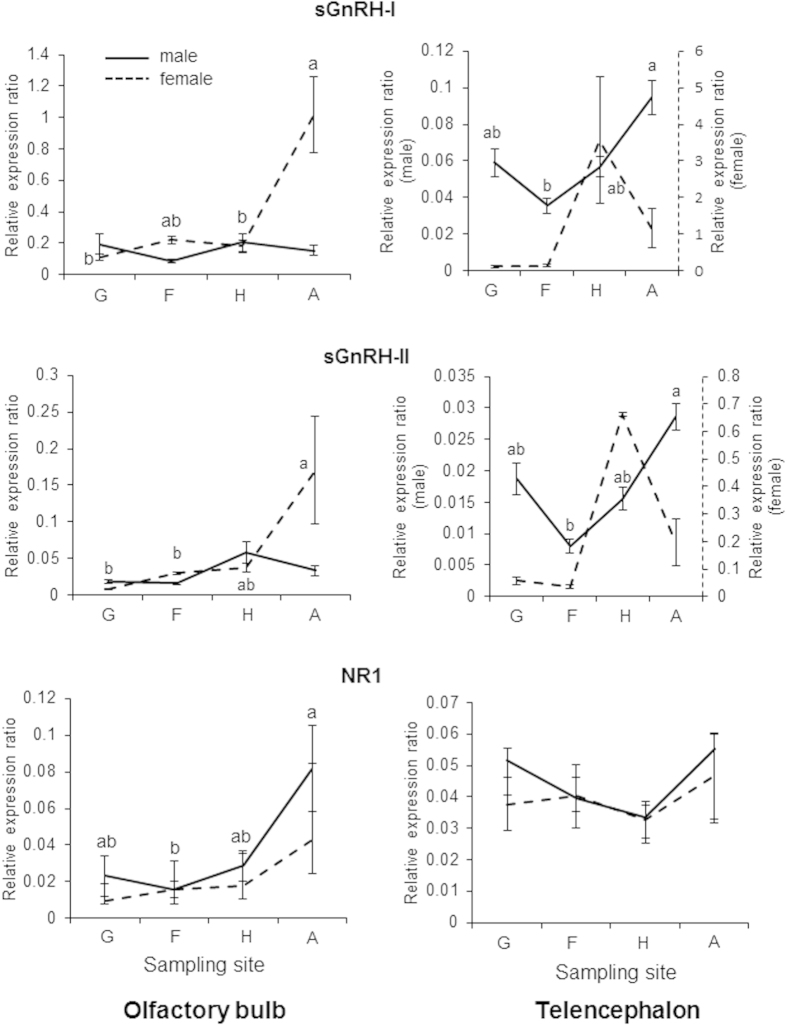
Changes in the profiles of salmon gonadotropin-releasing hormone (sGnRH) and the N-methyl-D-aspartate receptor essential subunit NR1 in the olfactory bulb and telencephalon of adult chum salmon during homing migration. Adult fish were sampled in the Bering Sea (Site **G**), Ishikari Bay (Site **F**), the Indian Waterwheel in the Chitose River (Site **H**), and the Chitose Hatchery (Site **A**). The gene expression ratios of sGnRH-I, sGnRH-II, and NR1 in the olfactory bulb and telencephalon of male and female adult chum salmon were normalized to the reference gene (β-actin), and the expression levels were compared using the relative Ct (ΔΔCT) method. Data are presented as the means ± SEM (N = 10). Different letters represent significant differences using the Steel-Dwass test (sGnRH-I and sGnRH-II in OB) and the Tukey-Kramer multiple comparison test (sGnRH-I, sGnRH-II in TE, and NR1) (*p* < 0.05).

**Figure 6:**
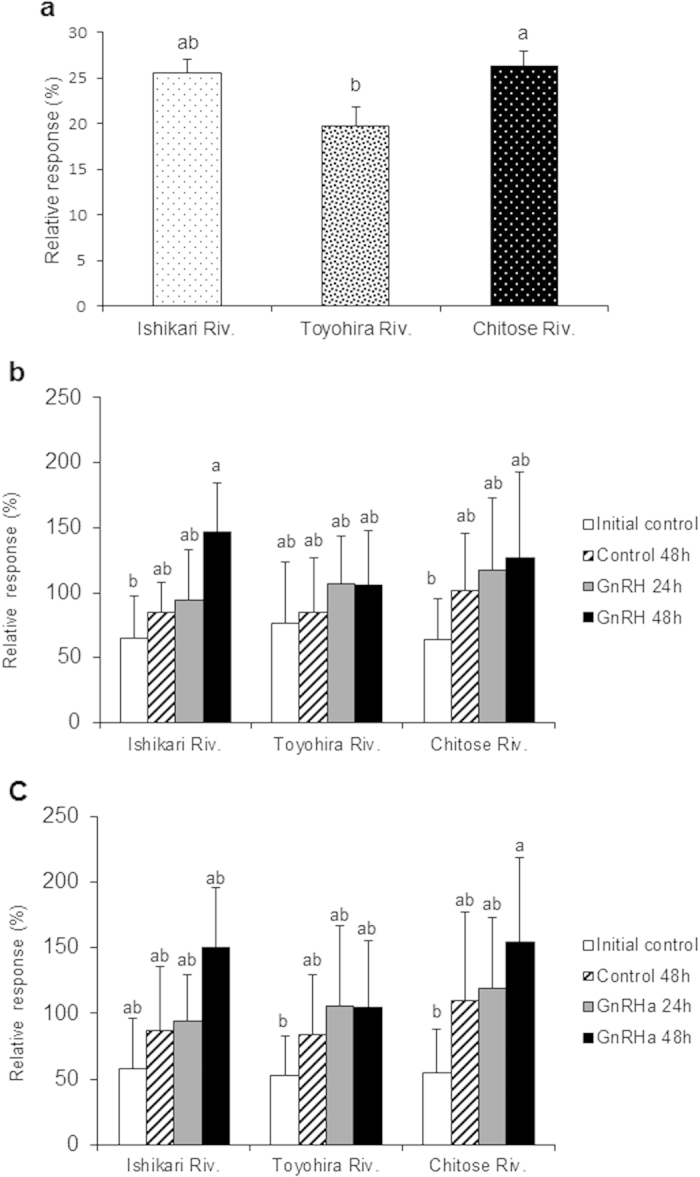
Electro-olfactogram (EOG) responses to waters from three streams in adult male chum salmon collected at the Indian Waterwheel (**a**), and effects of GnRH analogue implantation on the EOG response to three stream waters in adult male chum salmon collected from Ishikari Bay (b,c). **(a)** Adult fish were collected at the Indian Waterwheel (Site **H**). The relative EOG responses to the Ishikari River water, Chitose River water, and Toyohira River water are expressed as a proportion of the mean response to 0.1 mM L-Serine dissolved in distilled water. The values are means ± SEM of data obtained from seven adult fish. Different letters represent significant differences using one way ANOVA followed by the Dunnett test (*p* < 0.05). **(b)** Adult chum salmon including strays from another hatchery and **(c)** adult chum salmon originating from the Chitose Hatchery (confirmed by the otolith thermal marks) caught in Ishikari Bay (Site **F**) prior to entering to the river were reared in seawater at 13 °C using a refrigerator truck, implanted with either 300 μg (GnRH 24 h, 48 h) or 0 μg (Control 48 h) of GnRHa, and sampled at 0 (Initial control), 24, and 48 h. The relative EOG responses to the Ishikari River water, Chitose River water, and Toyohira River water are expressed as a proportion of the mean response to 0.1 mM L-serine dissolved in distilled water. Data are presented as the means ± SEM (N = 4–5). Different letters represent significant differences using two-way ANOVA followed by the Tukey-Kramer multiple comparison test (*p* < 0.05).

During the adult homing migration from the Bering Sea (Site **G**) in July to the Chitose Hatchery (Site **A**) in October, the mRNA expression levels of salmon gonadotropin-releasing hormone (sGnRH-I and II) and NR1 in the OB and TE appeared to increase, with some differences between males and females that might be related to sex-specific gonadal maturation^12^ (Fig. 5). Interestingly, sGnRH-II gene expression in the hypothalamus of males was also high in the Bering Sea (Supplementary Fig. 1). The EOG response of adult male chum salmon collected at Site **H** was significantly higher for both the Ishikari River and Chitose River, their home stream waters, than for the Toyohira River, a nearby tributary not on their migratory path (Fig. 6a). These data suggest that the olfactory memory retrieval abilities regarding natal stream odours increase during the upstream homing migration of adult chum salmon.

Prior work has shown that treatment with thyroid hormone can prompt olfactory cellular proliferation in coho salmon^24^ and the visual imprinting process in newly hatched chicks (*Gallus gallus domesticus*)^29^. In experiments with the oral administration of T4 to juvenile salmon, we found increased T4 and T3 levels in the whole body (Supplementary Fig. 2) and significant increases in NR1 gene expression in the whole brain for 4 days (Fig. 4b). Interestingly, T4 and T3 levels increased for 7 and 14 days, but no significant difference was observed in NR1 gene expression, suggesting that the critical period of imprinting is limited to less than 7 days.

The effects of implanting GnRH analogue (GnRHa), which can accelerate homing and gonadal maturation in sockeye salmon^25^,^26^, on the olfactory discrimination and olfactory memory retrieval of natal stream odours were studied using adult male chum salmon caught in Ishikari Bay (Site **F**) prior to entering the Ishikari River. GnRHa implantation increased the serum levels of 17α, 20β-dihydroxy-4-pregnen-3-one (DHP) after 48 h (Supplementary Fig. 3a), and tended to enhance NR1 gene expression in the OB after 24 and 48 h (Supplementary Fig. 3b). GnRHa implantation for 48 h significantly increased the relative EOG response to the Ishikari River water in adult male chum salmon, including straying fish from another hatchery (Fig. 6b). The relative EOG response of adult male chum salmon originating from the Chitose Hatchery (confirmed by otolith thermal marks) showed significant discrimination of Chitose River water (Fig. 6c), revealing that GnRHa stimulated the ability to discriminate natal stream odour.

## Discussion

The endocrine control of osmoregulation in relation to seawater adaptability has been intensively studied in teleost fish, including salmon^10^,^11^, and a relationship between the T4 surge and the onset of downstream migration has been reported in juvenile chum salmon^23^. However, there are few studies investigating the endocrine control mechanisms of olfactory imprinting during downstream migration in juvenile salmon. This study presents the first analysis of the gene expression profiles of whole-brain TRHa/b and upper head TSHβ in juvenile chum salmon, revealing that environmental changes due to the release from the hatchery to the natal stream significantly enhanced the TRHa/b expression levels and that the TSHβ expression levels and T4/T3 levels in the lower jaw increased significantly during downstream migration towards the sea.

Although the effects of salinity change on NR1 gene expression have been reported in juvenile chum salmon, demonstrating that NR1 expression increases with increased salinity^22^, there are no data addressing how NR1 is involved in olfactory imprinting during downstream migration. In this study, NR1 expression levels showed a significant first peak correlated with larval brain development in the hatchery^27^, a gradual significant increase immediately after a significant TRHa/b expression surge at Site **C**, and a second significant peak at the mouth of their natal river (Site **E**). The results of *in situ* hybridization confirmed that NR1 mRNA localization increased prominently in the ventral TE at Site **C** compared to the hatchery immediately before release into the river. Moreover, the oral administration of T4 significantly increased NR1 expression levels for 4 days.

Natal stream odour discrimination abilities were examined using the EOG response to stream water odours, revealing significantly greater discrimination ability for the Ishikari River (home stream) water in juvenile chum salmon collected at Sites **B** and **D** during downstream migration compared to those at the hatchery. The olfactory memory formation capacity for stream odours likely develop when juvenile chum salmon encounter different streams during their downstream migration, and these capabilities are clearly linked to NR1 and BPT hormones. Cumulatively, our data suggest that during the initiation of the downstream migration of juvenile chum salmon, the environmental changes involved in the release into the river may induce the expression of the BPT hormones, which then stimulate the upregulation of NR1, enhancing the olfactory memory formation capability.

The homing migration of adult chum salmon is closely related to gonadal maturation, which is regulated mainly by the brain-pituitary-gonad hormones. In particular, sGnRH in the olfactory system, terminal nerve, and preoptic area is believed to play brain region-dependent leading roles in salmon homing migration^9^,^12^. This study presents a high level of sGnRH-II gene expression in the hypothalamus in the Bering Sea, suggesting a correlation with the onset of gonadal maturation as well as the start of homing migration. During upstream homing migration from Ishikari Bay (Site **F**) to the natal hatchery (Site **A**), the gene expressions levels of sGnRH-I/II and NR1 in the OB and TE showed some sexually differentiated patterns, likely due to sex-specific gonadal maturation^12^.

Adult male chum salmon collected at Site **H** showed significantly higher EOG responses to their natal stream water than to non-natal stream water. GnRHa implantation in adult male chum salmon caught in Site **F** prior to entering their natal stream produced different EOG responses whether including straying fish from another hatchery or only originating from the natal hatchery. The relative EOG response of adult male chum salmon originating from the Chitose Hatchery showed significant discrimination of the Chitose River water, but that including straying fish only showed significant discrimination of the Ishikari River water. These data suggest that straying fish and accurately homing fish show different olfactory discrimination ability for their natal stream waters.

Our data on adult homing migration suggest that the activation of sGnRHI/II may cause both the onset of homing migration and the upregulation of NR1, facilitating olfactory memory retrieval as well as natal stream odour discrimination during upstream migration. This study demonstrates that the gene expression levels of NR1 in the brain are a useful molecular marker to clarify the olfactory imprinting and homing abilities of both juvenile and adult chum salmon. While measurement of NR1 transcription is a good first step, it is the protein that will be directly involved in memory formation, and this has not been measured in the data presented. It is unclear whether the relatively small changes in transcription (especially in the downstream migratory stage) result in increased protein levels. Further biochemical and molecular biological studies investigating the protein levels and the gene expression profiles of NR1 and NR2A-D with treatments of NMDAR antagonist will be able to reveal olfactory long-term potentiation in salmon brain.

These results have important ramifications for salmon conservation and production. Straying, or lack of appropriate homing, is present in all migratory populations to varying degrees, and its expression may be either positive (leading to dispersal) or negative (yielding loss of spawners in a population^30^). Understanding homing mechanisms is critical to the management of straying potential. This application may also be useful for salmon propagation. In Japan, chum salmon is mainly propagated through a hatchery ranching programme that is believed to be one of the most successful fish propagation systems in the world; however, production has decreased in the aftermath of the 2011 Tohoku earthquake and tsunami^31^. Recently, a new embryonic imprinting paradigm for hatchery programme has been introduced in the USA^32^. Because T4 treatment may be effective in increasing olfactory imprinting abilities in salmon, we suggest that the salmon conservation and propagation programme incorporate treatment with biologically active substances to enhance the BPT hormone production to improve the olfactory imprinting capability of juvenile salmon and thus decrease straying and support long-term sustainable salmon conservation and propagation.

## Methods

### Fish Collection

Juvenile chum salmon were captured in the river using cast nets (Sites **B**, **C**, **D**, and **E** in Fig. 1), and in Ishikari Bay using surface trawling nets from two boats (Site **F**). Adult chum salmon were captured with fish traps in the Chitose River at the Chitose Hatchery (Site **A**) and the Indian Waterwheel (Site **H**), with set nets in Ishikari Bay (Site **F**), and with longlines as part of the R.V. Hokko-Maru 2013 summer cruise in the Bering Sea (Site **G**). All fish were collected in accordance with the animal care guidelines of Hokkaido University.

Underyearling juvenile chum salmon reared at the Chitose Hatchery (Site **A**), Hokkaido National Fisheries Research Institute, Chitose, Hokkaido, Japan were sampled monthly from January (**A1**) to April (**A4**) of 2013. After release into the Chitose River, they were sampled at the second bridge of the Chitose (Site **B**), the Chitose River in the Kamaka Ward (Site **C**), the confluence point of the Chitose and Yubari Rivers (Site **D**), the mouth of the Ishikari River (Site **E**), and Ishikari Bay (Site **F**) in April 2013. The fork length (FL) and body weight (BW) of the fish are provided in Supplementary Table 1a.

For the EOG experiment on juvenile chum salmon during downstream migration in April 2015, three fish were used at each sampling point: the Chitose Hatchery (Site **A**), the second bridge of the Chitose (Site **B**), the Chitose River in the Kamaka Ward (Site **C**), the confluence point of the Chitose and Yubari Rivers (Site **D**), the mouth of the Ishikari River (Site **E**), and Ishikari Bay (Site **F**). The FL and BW of the fish are provided in Supplementary Table 1b.

For the oral T4 administration experiment, juvenile chum salmon reared at the Chitose Hatchery were transferred and reared in spring water at the Sapporo Salmon Museum, Sapporo, Hokkaido, Japan, in March 2015. They were fed pellets (Salmon juvenile No. 1, Marubeni Nisshin Feed Co. LTD, Tokyo, Japan) containing T4 (Sigma, St. Louis, Missouri, USA) for 14 days. T4 (100 mg) was dissolved into 220 μl of 4 M NaOH, added to 3.5 ml of ethanol, sprayed over 50 g of pellets, and dried, for a T4 final concentration of 2 mg/g pellet. Fish were sampled at day 0 (Initial control), 1 (Control and Experiment 1d), 4 (Control and Experiment 4d), 7 (Control and Experiment 7d), and 14 (Control and Experiment 14 d). The FL and BW of the fish are provided in Supplementary Table 1c.

Adult chum salmon (average of 4 years old) of both sexes were sampled in the Bering Sea (54° 00′ N, 175° 00′ E) (Site **G**) in late July 2013, and in Ishikari Bay (Site **F**), the Indian Waterwheel (Site **H**), and the Chitose Hatchery (Site **A**) in October 2013. The FL, BW and GSI (gonad weight/body weight × 100) of the fish are provided in Supplementary Table 2a.

For the EOG experiment on adult male chum salmon during homing, seven fish were collected at the Indian Waterwheel (Site **H**) in October 2014. We only use adult male chum salmon due to limited experimental time, but we have previously reported no significant differences in EOG response between male and female salmon^33^. The FL, BW and GSI of the fish are provided in Supplementary Table 2b.

For the GnRHa implantation experiment, adult male chum salmon caught in Ishikari Bay (Fig. 1; Site **F**) prior to entering the Ishikari River were transferred to the Sapporo Salmon Museum and reared in seawater from Ishikari Bay maintained at 13 °C using a refrigerator truck in October 2014. They received a 2 mm intramuscular capsule implant via a 17 gauge needle; the capsule contained 300 μg of GnRHa ([D-Ala^6^, Pro^5^NET]-GnRH) or 0 μg of GnRHa (control) in an ethylene-vinyl acetate copolymer matrix^34^. Sampling and EOG were completed at 0 h (Initial control), 24 h (GnRHa 24 h), and 48 h (GnRHa 48 h and Control 48 h). We only used adult male chum salmon due to limited experimental facilities. Although there were some differences in gonadal maturation with GnRHa implantation between male and female salmon, the effects of GnRHa implantation were nearly identical for the two sexes^25^,^26^. The FL, BW and GSI of the fish are provided in Supplementary Table 2c.

For sampling juvenile chum salmon, fish were anesthetized with 0.05% FA-100 (4-allyl-2-methoxyphenol; DS Pharma Animal Health, Osaka, Japan). To measure TRHa/b and NR1 gene expression, fish were decapitated, and the whole brain was removed. To measure TSHβ gene expression, fish were decapitated and the upper head, which contains the pituitary gland, was removed. To measure T4 and T3 levels, we used the lower jaw, containing the thyroid gland, for some fish and the entire body without the head for other fish. The samples were frozen in liquid nitrogen and stored at −80 °C.

For sampling adult chum salmon, fish were quickly immobilized by a gentle hit on the snout and then immediately decapitated, after which the brain was removed. Three regions of the brains were collected: the olfactory bulb (OB), telencephalon (TE), and hypothalamus (HT). To measure serum DHP levels, blood was collected from the caudal vasculature and centrifuged at 1,200 g for 15 min at 4 °C. The resulting brain and serum samples were frozen in liquid nitrogen and stored at −80 °C.

### Recording of the Electro-Olfactogram (EOG) Response

The EOG response^35^,^36^ was measured using 3–7 randomly selected fish, and the magnitude of the response is expressed as a percentage of the response to 0.1 mM L-serine (Ser) dissolved in distilled water. Fish were immobilized with an intramuscular injection of gallamine triethiodide (3 μg/g). Gills were aerated through the mouth with an aerated solution of clove oil (0.005%), which was not allowed to contact the olfactory rosettes. The responsive properties of the olfactory receptor cells were recorded using a pair of glass microelectrodes filled with 2.5% agar-saline and bridged with silver wire. With the aid of a stereomicroscope and micromanipulators, an odorant perfusion tube was gently inserted into the in-current passage of the nose, and the recording microelectrode was inserted through the out-current passage and positioned above the midline of the rosette at the base of the posterior-most lamella. A reference microelectrode was placed on the head, and a separate ground electrode was clipped to the tail of the fish. The differential electrical signal was amplified 500-fold and low-pass filtered at 100 Hz by a direct-current amplifier (A-M System, Carlsberg, Washington, USA). The signals were digitized at 10 samples per second using the PicoScope data-acquisition software (Pico Technology Ltd., St. Neots, UK), and the signal amplitudes were measured in mV. After electrode placement, the olfactory rosettes were rinsed for 30 min with the Sapporo Salmon Museum spring water at a rate of 1 ml/min. Each odour was then pulsed three times into the Sapporo Salmon Museum spring water for 10 s at 150 s intervals. EOGs were recorded in response to 0.1 mM Ser dissolved in distilled water (standard), the Ishikari River water (Site **E**), the Toyohira River water (Site **I**), or the Chitose River water (Site **B**). The amplitude of the EOG response was quantified by the displacement of the odour-evoked peak relative to the pre-stimulus electrical baseline. The magnitude of the response is expressed as the mean percentage of the response to 0.1 mM Ser ± SEM.

### Extraction of Total RNA and cDNA Synthesis

Total RNA was extracted from each sample using TRIzol (Life Technologies, Carlsbad, California, USA) according to the manufacturer’s protocol. The quality of RNA was measured at A260 nm and the purity was assessed using the A260/A280 nm ratio (Amersham Biosciences, Tokyo, Japan). Only RNAs with A260/A280 nm ratios of 1.6–2.0 were used for cDNA synthesis. Reverse transcription was performed on 0.5 μg of total RNA using a ReverTra Ace® qPCR RT Master Mix with gDNA Remover (TOYOBO, Osaka, Japan) according to the manufacturer’s protocol.

### Real-Time Quantitative PCR

Real-time PCR was used to detect the expression levels of TRHa/b, TSHβ, sGnRH-I/II, and NR1 mRNA in all samples. The β-actin mRNA expression levels were also measured in all samples as a housekeeping gene. The following primer sets were designed: TRHa qPCRF/TRHa qPCRR, TRHb qPCRF/ TRHb qPCRR, TSHβ qPCRF/ TSHβ qPCRR, sGnRH-I qPCRF/sGnRH-I qPCRR, sGnRH-II qPCRF/sGnRH-II qPCRR, NR1 qPCRF/NR1 qPCRR, and β-actin qPCRF/β-actin qPCRR (Supplementary Table 3). Quantitative PCR was performed using a real-time monitoring thermal cycler, MX3000P (STRATAGENE, La Jolla, California, USA). PCR reactions were conducted in a volume of 20 μl containing 5 μl of THUNDERBIRD SYBR qPCR Mix (TOYOBO), 0.04 μl of 50 × ROX, 0.06 μl of 100 μM primers, 13.84 μl of nuclease-free water, and 1 μl of each cDNA template, according to the manufacturer’s protocol. PCR amplifications were performed in duplicate wells, using the following cycling parameters: a single exposure of 95 °C for 5 min followed by 40 cycles of 15 s at 95 °C, 15 s at 55 °C to 60 °C (depending on the annealing temperature for the primer), and 30 s at 72 °C, with a final program-ending temperature ramp from 55 °C to 95 °C for a melt curve analysis. In each assay, samples were run together with non-template controls. All samples and standards were run in duplicates. The amplification efficiency of each primer pair was within 90–100%. TRHa/b, TSHβ, sGnRH-I/II, and NR1 mRNAs were normalized to the reference gene (β-actin), and expression levels were compared using the relative Ct (ΔΔCT) method.

### *In situ* Hybridization

Juvenile chum salmon were sampled at the Chitose Hatchery (Site **A**) and after release into the Chitose River at the Kamaka site downstream on the Chitose River (Site **C**) in April 2013. The FL and BW of the fish are provided in Supplementary Table 1d. Fish were anesthetized with 0.05% FA-100. After decapitation, the heads were immediately frozen in powdered dry ice. The head sections were cut towards the direction of the coronal plane into 20 μm-thick slices with a cryostat (CM1900; Leica, Solms, Germany) and mounted on silane-coated glass slides. For hybridization, digoxigenin (DIG)-labeled sense and antisense cRNA probes were prepared to detect NR1 mRNA. The primer set NR1 qPCRF/ NR1 qPCRR (Supplementary Table 4) was designed to amplify 451 bp-long fragments. Then, cDNA fragments of NR1 were sub-cloned into the pGEM^®^-T Easy Vector (Promega, Tokyo, Japan). Using the linearized plasmid, *in vitro* transcription was performed using T7 or SP6 RNA polymerase. Sections were treated with the following incubation steps: fixation with 4% paraformaldehyde-PB (pH 7.2) for 10 min, washing in PBS (pH 7.2) for 10 min, acetylation with 0.25% acetic anhydride in 0.1 M triethanolamine-HCl (pH 8.0) for 10 min, and pre-hybridization for 30 min in a hybridization buffer (50% formaldehyde, 50 mM Tris-HCl (pH 7.5), 0.02% Ficoll, 0.02% polyvinylpyrrolidine, 0.02% bovine serum albumin, 0.6 M NaCl, 200 μg/ml of tRNA, 1 mM EDTA, and 10% dextran sulfate). Hybridization was performed at 63.5 °C for 12 h in the hybridization buffer supplemented with sense or antisense cRNA probes at a dilution of 1:1,000. Post-hybridization washing was performed successively at 61 °C with 5 × SSC (1 × SSC: 150 mM NaCl and 15 mM sodium citrate) for 30 min, 4 × SSC containing 50% formamide for 40 min, 2 × SSC containing 50% formamide for 40 min, and 0.1 × SSC for 30 min. Sections were dipped at room temperature in 0.1 × SSC for 15 min, NTE buffer (0.5 M NaCl, 0.01 M Tris-HCl, pH 8.0, and 5 mM EDTA) for 20 min, 20 mM iodoacetamide in NTE buffer for 20 min, and TNT buffer (0.1 M Tris-HCl, pH 7.4, and 0.15 M NaCl) for 20 min. For immunofluorescent detection, sections were blocked in TNT buffer for 10 min, DIG blocking solution (10% blocking reagent (Roche, Basel, Switzerland): normal sheep serum: TNT buffer = 1:1:8) for 30 min, and 0.5% TSA blocking reagent (PerkinElmer, Waltham, Massachusetts, USA) in TNT buffer for 30 min. They were subjected to detection by a DIG-labeled cRNA probe using peroxidase-conjugated anti-DIG antibody in DIG blocking solution (1:500, Roche, Basel, Switzerland) for 1 h with the Cy3-TSA Plus amplification kit (PerkinElmer) and TNT buffer overnight. On the following day, for counterstaining, sections were labeled with 0.05% NeuroTrace® 500/525 green fluorescent Nissl stain (Invitrogen, Carlsbad, California, USA) in PBS (pH 7.2) for 10 min followed by PBS (pH 7.2) for 20 min. All of the washing and incubation solutions contained 0.0005% Tween 20. Images were taken with an FV1000 laser-scanning confocal microscope (Olympus, Tokyo, Japan).

### T4 and T3 Levels

The total T4 and T3 levels in the lower jaw or whole body were measured by enzyme-linked immunosorbent assay (ELISA)^37^. Samples (0.2–2.0 g) were homogenized in 1.0 ml of ice-cold methanol. The homogenate was decanted and kept on ice. The tube used in homogenization and the blade were rinsed twice with 100 μl of ice-cold methanol. These rinses were added to the homogenate. After centrifugation at 1,200 g for 15 min at 4 °C, the supernatant was stored and the precipitate was re-extracted with 200 μl of methanol. After centrifugation at 1,200 g for 15 min at 4 °C, the supernatant was added to the previous supernatant. The combined supernatant was then vacuum-dried at 37 °C. The residue was re-dissolved in a mixture of 50 μl of methanol and 50 μl of 0.11 M barbital buffer (pH 8.6) using a Vortex mixer for approximately 10 s and then mixed with 200 μl of chloroform using a Vortex mixer for approximately 10 s. After centrifugation at 1,200 g for 10 min at 4 °C, the upper polar layer, which contained the majority of the T4 and T3, was transferred to a glass tube with a Pasteur pipet. At this stage, the lipids and lipoproteins remained in the nonpolar layer. The upper layer was vacuum-dried overnight at 37 °C. The residue was re-dissolved in 300 μl of 0.11 M barbital buffer (pH 8.6). The levels of T4 and T3 in the lower jaw or whole body were measured with the T4 AccuBind® ELISA and the T3 AccuBind® ELISA (Monobind, Lake Forest, California, USA), respectively, according to the manufacturer’s protocol.

### Serum DHP levels

Serum DHP levels were measured using a time-resolved fluoroimmunoassay (TR-FIA)^38^. In brief, 0.05 μg/ml DHP-BSA conjugate was immobilized into 96-well microtitre plates (Wallac Oy, Turku, Finland). Each extracted sample (50 μl) and DHP antibody (150 μl) were dispensed into wells. After reacting for 18 h at 4 °C, Eu-labelled IgG (DELFIA Eu-N1 labeled anti-rabbit IgG antibody (goat), PerkinElmer) was placed into each well, incubated for 1 h at room temperature, and then thoroughly washed to remove any unbound Eu-labelled IgG. The fluorescence intensity from the dissociated Eu was then measured using a microplate reader (Infinite F500, Tecan, Männedorf, Switzerland). The intra- and inter-assay coefficients of variation for DHP were 7.5% and 6.1%, respectively. The displacement curve of the extracted sample was paralleled by the DHP standard curve.

### Statistics

Differences were considered significant at *p* < 0.05. For the downstream migration of juvenile chum salmon, statistical significance was determined using the Tukey-Kramer multiple comparison test and Scheffe’s F test. For the EOG experiments in juvenile and adult chum salmon, statistical significance was determined using one-way analysis of variance (ANOVA) followed by the Dunnett test and two-way ANOVA followed by the Tukey-Kramer multiple comparison test. For the T4 administration experiment on juvenile chum salmon, statistical significance was determined using the Steel-Dwass test and Scheffe’s F test. For adult chum salmon homing migration, statistical significance was determined using the Steel-Dwass test, Tukey-Kramer multiple comparison test, and Scheffe’s F test. For the GnRHa implantation experiment on adult chum salmon, statistical significance was determined using the Steel-Dwass test and two-way ANOVA followed by the Tukey-Kramer multiple comparison test.

### Ethics statement

This study (16-8) was approved to be conducted under the control of the committee following the “Guide for Care and Use of Laboratory Animals in Field Science Center for Northern Biosphere, Hokkaido University”, Japanese Governmental Law (No. 105) and Notification (No. 6).

## Additional Information

**How to cite this article**: Ueda, H. *et al.* Involvement of hormones in olfactory imprinting and homing in chum salmon. *Sci. Rep.* **6**, 21102; doi: 10.1038/srep21102 (2016).

## Supplementary Material

Supplementary Information
