# Erratum: Involvement of hormones in olfactory imprinting and homing in chum salmon

**DOI:** 10.1038/srep24405

**Published:** 2016-04-22

**Authors:** Hiroshi Ueda, Shingo Nakamura, Taro Nakamura, Kaoru Inada, Takashi Okubo, Naohiro Furukawa, Reiichi Murakami, Shigeo Tsuchida, Yonathan Zohar, Kotaro Konno, Masahiko Watanabe

Scientific Reports 6: Article number: 21102; 10.1038/srep21102published online: 02162016; updated: 04222016

In the Supplementary Information file originally published with this Article, the lower part of Fig. S1 ‘sGnRH-II’ was incorrectly labelled ‘GnRH-II’. This error has been corrected in the Supplementary Information that now accompanies the Article.

