# Supplementary material for: Involvement of hormones in olfactory imprinting and homing in chum salmon
Source: Sci Rep. 2016 Feb 16;6:21102. doi: 10.1038/srep21102 (PMC4754751; doi:10.1038/srep21102)
Supplement: Supplementary Information [file srep21102-s1.pdf]

Involvement of hormones in olfactory imprinting and homing in chum salmon

Hiroshi Ueda[1, 2]*, Shingo Nakamura[2], Taro Nakamura[2], Kaoru Inada[3], Takashi Ookuβo[2], Naohiro Furukawa[2], Reiichi Murakami[2], Shigeo Tsuchida[2], Yonathan Zohar[4], Kotaro Konno[5] & Masahiko Watanabe[5]

[1]Field Science Center for Northern Biosphere, Hokkaido University, Sapporo 060-0809, Japan.

[2]Division of Biosphere Science, Graduate School of Environmental Science, Hokkaido University, Sapporo 060-0809, Japan.

[3]Department of Natural History Sciences, Graduate School of Science, Hokkaido University, Sapporo 060-0810, Japan.

[4]Department of Marine Biotechnology, Institute of Marine and Environmental Technology, University of Maryland, Baltimore, MD 21202, USA.

[5]Department of Anatomy, Graduate School of Medicine, Hokkaido University, Sapporo 060-8638, Japan.

*Correspondence and requests for materials should be addressed to H.U. (e-mail: hueda@fsc.hokudai.ac.jp)

Supplementary information

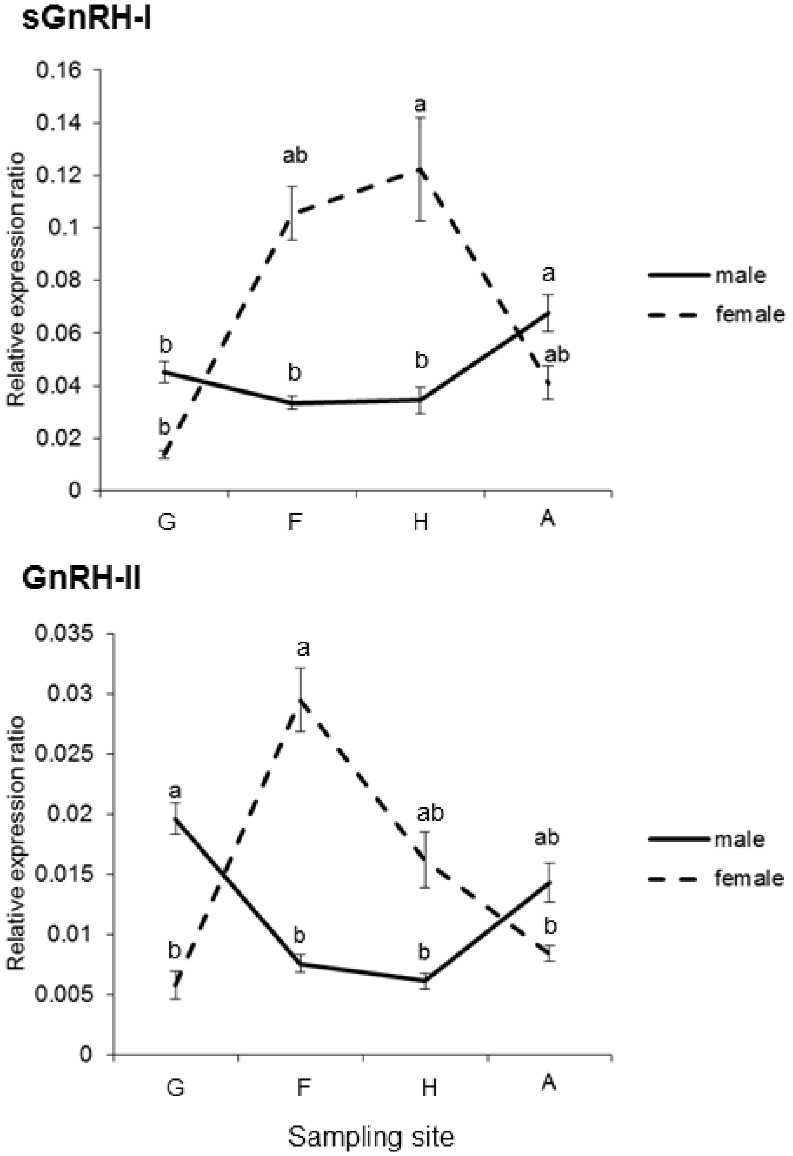

Fig. S1. **Changes in profiles of salmon gonadotropin-releasing hormone (sGnRH) in the hypothalamus of adult chum salmon during homing migration.** Fish were sampled in the Bering Sea (**G**), Ishikari Bay (**F**), the Indian Waterwheel (**H**), and the Chitose Hatchery (**A**). The gene expression ratios of sGnRH-I and sGnRH-II in the hypothalamus of male and female adult chum salmon were normalized to the reference gene (β-actin), and expression levels were compared using the relative Ct (ΔΔCT) method. Data are presented as the means ± SEM (N=3-5). Different letters represent significant differences using Scheffe’s F test (sGnRH-I) and the Steel-Dwass test (sGnRH-II) (p<0.05).

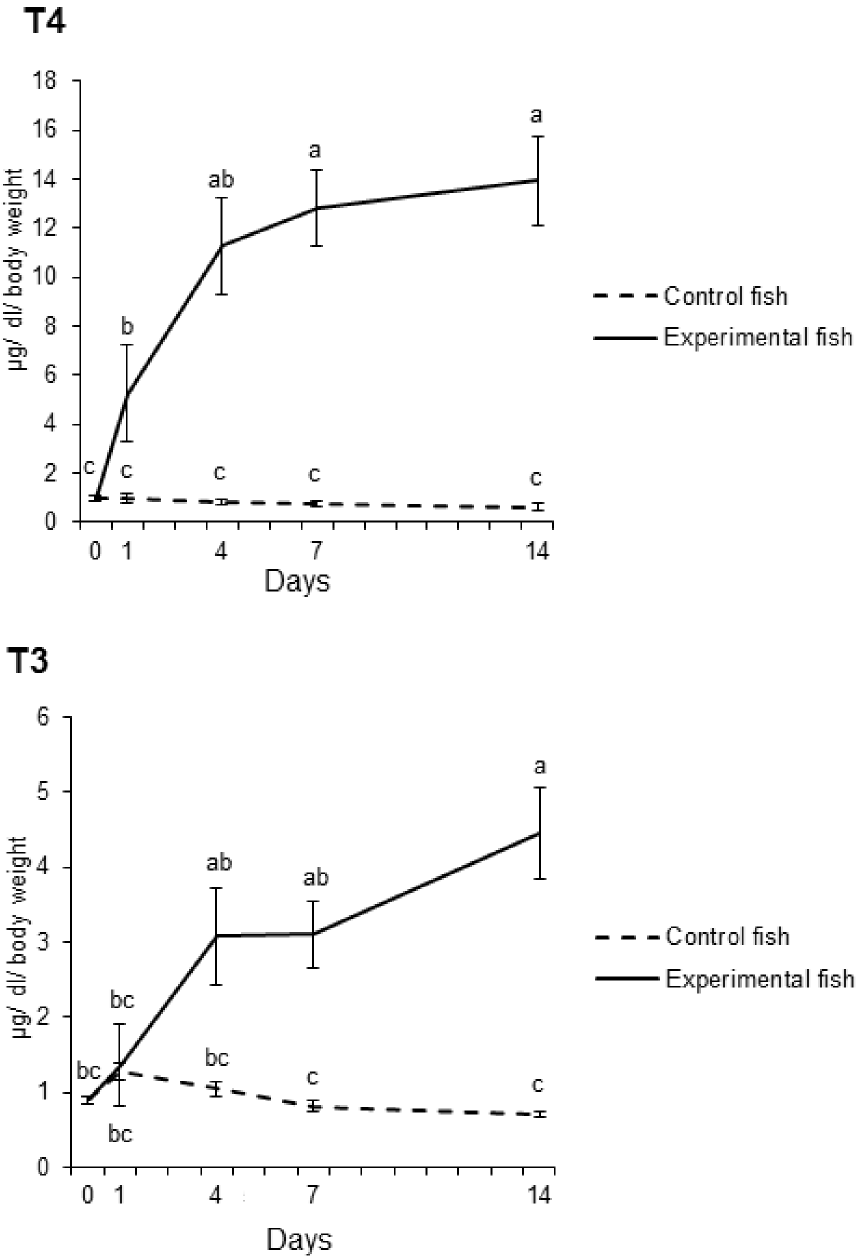

Fig. S2. Effects of oral thyroxine (T4) administration on T4 and triiodothyronine (T3) levels in the whole body of juvenile chum salmon. Juvenile chum salmon reared in the Chitose Hatchery were transferred to the Sapporo Salmon Museum, Sapporo, Hokkaido, Japan, fed pellets containing 2 mg/g T4 (Experiment fish) or pellets without T4 (Control fish) for 14 days, and sampled at days 0 (Initial control) 1, 4, 7, and 14. The levels of T4 and T3 in the whole body are expressed in μg/dl/body weight. Data are presented as the means ± SEM (N=7-8). Different letters represent significant differences using Scheffe's F test (p<0.05).

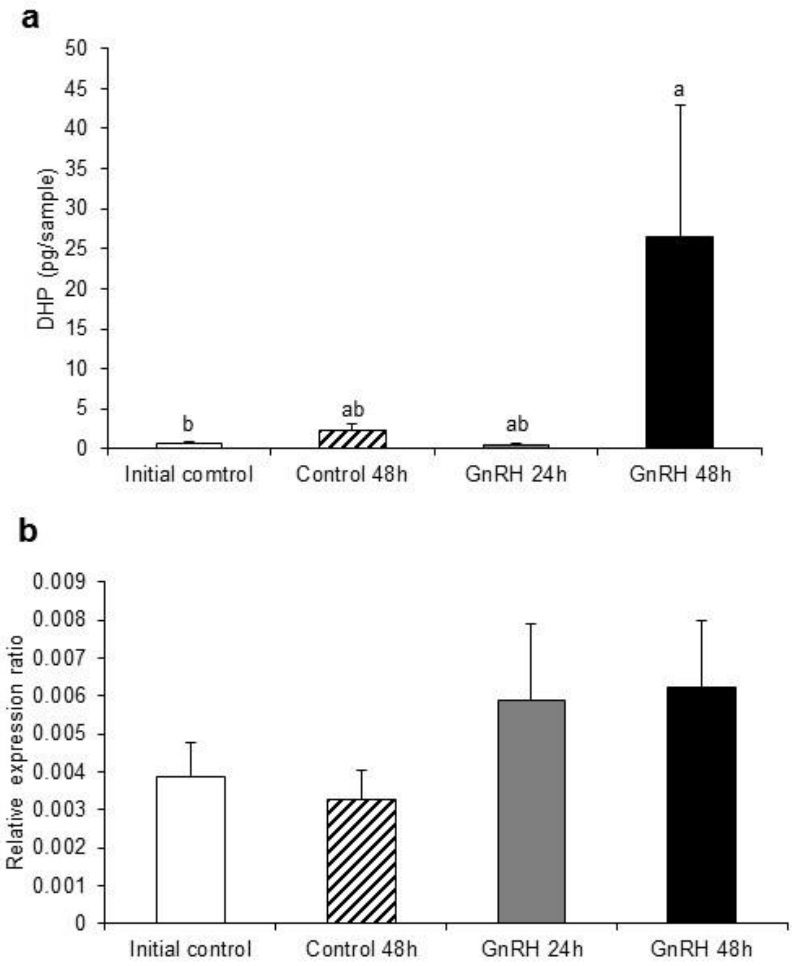

Fig. S3. Effects of GnRH analogue implantation on serum 17α,20β-dihydroxy-4-pregnen-3-one (DHP) levels (a) and the gene expression levels of the N-methyl-D-aspartate receptor essential subunit NR1 in the olfactory bulb of adult male chum salmon collected in the Ishikari Bay (b). Adult male chum salmon caught in the Ishikari Bay (Fig. 1; Site **F**) prior to entering to the Ishikari River were transferred to the Sapporo Salmon Museum and reared in seawater maintained at 13°C using a refrigerator truck. Experimental and control fish were implanted with a capsule containing 300 μg or 0 μg of GnRHa, respectively, and were sampled at 0 h (Initial control), 24 h (GnRH 24 h), and 48 h (Control and GnRH 48 h). Data are presented as the means ± SEM (N=4-5). Different letters represent significant differences using the Steel-Dwass test (p<0.05).

Table S1. Fork length (FL) and body weight (BW) of juvenile chum salmon. (a) Fish were sampled during downstream migration from the Chitose Hatchery (Fig. 1; Site **A**: **A1**, January; **A2**, February; **A3**, March; **A4**, April), the second bridge of the Chitose River (**B**), the Chitose River at Kamaka Ward (**C**), the confluence point of the Chitose and Yubari Rivers (**D**), the mouth of the Ishikari River (**E**), and Ishikari Bay (**F**). N=10-15. **(b)** Fish used for the electro-olfactogram (EOG) experiment. N=3. **(c)** Fish used for the oral T4 administration experiment, and sampled on days 0 (Initial control), 1 (Control and Experiment 1d), 4 (Control and Experiment 4 d), 7 (Control and Experiment 7 d), and 14 (Control and Experiment 14 d). N=7-8. **(d)** Fish used for the *in situ* hybridization experiment from the Chitose Hatchery (A in April; **A4**) and the Chitose River in Kamaka Ward (**C**). N=3. All data are presented as the means ± SEM.

	Sampling site/ Treatment	FL(cm)	BW(g)
**a**	A1	3.55±0.02	0.35±0.01
	A2	3.85±0.02	0.42±0.01
	A3	4.08±0.03	0.58±0.02
	A4	4.85±0.06	0.95±0.03
	B	4.88±0.04	0.84±0.02
	C	4.87±0.04	0.90±0.33
	D	4.30±0.12	0.57±0.06
	E	4.58±0.08	0.69±0.05
	F	5.13±0.16	1.46±0.13
**b**	A	5.33±0.09	1.05±0.01
	B	5.10±0.36	0.96±0.09
	C	4.43±0.07	0.64±0.05
	D	5.57±0.24	1.35±0.16
	E	5.27±0.28	1.11±0.16
	F	4.90±0.17	0.90±0.07
**c**	Initial control	6.49±0.09	1.84±0.09
	Control 1 d	6.39±0.12	1.89±0.11
	Experiment 1 d	6.45±0.11	1.95±0.09
	Control 4 d	6.45±0.13	2.00±0.12
	Experiment 4 d	6.60±0.12	1.99±0.10
	Control 7 d	6.60±0.10	1.97±0.01
	Experiment 7 d	6.62±0.06	1.88±0.06
	Control 14 d	6.47±0.12	1.83±0.11
	Experiment 14 d	6.56±0.15	1.97±0.14
**d**	A4	4.86±0.25	0.96±0.11
	C	4.70±0.17	0.82±0.10

Table S2. Fork length (FL), body weight (BW) and gonadosomatic index (GSI) of adult chum salmon. (a) Fish were sampled during homing migration from the Bering Sea (**G**), Ishikari Bay (**F**), the Indian Waterwheel (**H**), and the Chitose Hatchery (**A**). N=10. **(b)** Fish used for the electro-olfactogram experiment collected at the Indian Waterwheel (**H**). N=7. **(c)** Fish used for the GnRH analogue implantation experiment and sampled at 0 h (Initial control), 24 h (GnRH 24 h), and 48 h (Control and GnRH 48 h). (N=4-5). All data are presented as the means ± SEM.

	Sampling site/ Treatment	Male			Female		
		FL(cm)	BW(kg)	GSI (%)	FL(cm)	BW(kg)	GSI (%)
**a**	G	49.8±2.79	1.96±0.18	0.12±0.03	53.6±1.60	2.07±0.17	1.84±0.50
	F	58.8±1.95	2.30±0.22	5.26±0.28	63.9±0.60	2.75±0.11	16.1±0.89
	H	67.1±2.28	3.19±0.29	4.13±0.27	64.8±0.44	2.75±0.14	20.40±0.52
	A	72.4±1.12	4.17±0.17	3.37±0.19	68.1±0.75	2.73±0.11	2.27±0.23*
**b**	H	68.2±1.97	3.48±0.22	4.20±0.30			
**c**	Initial control	62.8±2.04	3.23±0.29	4.43±0.27			
	Control 48 h	60.7±1.69	2.15±0.20	5.28±0.14			
	GnRH 24 h	60.4±1.26	2.22±0.14	5.70±0.42			
	GnRH 48 h	60.9±1.26	2.52±0.21	5.33±0.37			

*Post-ovulatory stage.

Table S3. Specific primers for chum salmon β-actin, NR1, TRHa/b, TSHβ, and sGnRH-I/II genes for amplification via real-time quantitative PCR.

Genes	Primer	DNA sequences
β-actin	Forward	5`-ATTTGGCATCACACCTTCT-3`
	Reverse	5`-TTCTCCCTGTTGGCTTTG-3`
NR1	Forward	5`-AGGAGTGTTCATGCTGGTGG-3`
	Reverse	5`-GTCGCTTGTAGGCGATCTCA-3`
TRHa	Forward	5`-TGGCGAGGTTCAGAAAAGAC-3`
	Reverse	5`-CACTTAGAAATGCGGGGTTC-3`
TRHb	Forward	5`-ATGAGGCATTGTCCTCTCAG-3`
	Reverse	5`-TGCTGTCTTTTCTGGACCTC-3`
TSHβ	Forward	5`-TCATATGTGTGCCACGGACTAC-3`
	Reverse	5`-TGGTCGTATTGATGGCCACG-3`
sGnRH-Ⅰ	Forward	5`-GACTGAGACCATATGAGTCATGTC-3`
	Reverse	5`-TTACAATTTTACAGGAGGTTAGTGTTTC-3`
sGnRH-Ⅱ	Forward	5`-GAGACTGAGACCATATGATGTAATATTG-3`
	Reverse	5`-GAATGGTCCATCATTGATGTTG-3`

Table S4. Specific primers for chum salmon NR1 gene amplification for *in situ* hybridization.

Gene	Primer	DNA sequences
NR1	Forward	5`-GGAGGAGAGGGAAACAAAGG-3`
	Reverse	5`-AGTGAGGCCCTCTGGGTACT-3`
